# The Utility of Proton Beam Therapy with Concurrent Chemotherapy for the Treatment of Esophageal Cancers

**DOI:** 10.3390/cancers3044090

**Published:** 2011-10-28

**Authors:** Steven H. Lin

**Affiliations:** Thoracic Radiation Oncology Group, Department of Radiation Oncology, The University of Texas MD Anderson Cancer Center, Houston, TX 77030, USA; E-Mail: shlin@mdanderson.org; Tel.: +1-713-563-8490; Fax: +1-713-563-2366

**Keywords:** esophageal cancer, proton beam, IMRT, chemotherapy

## Abstract

The standard of care for the management of locally advanced esophageal cancers in the United States is chemotherapy combined with radiation, either definitively, or for those who could tolerate surgery, preoperatively before esophagectomy. Although the appropriate radiation dose remains somewhat controversial, the quality of the radiation delivery is critical for the treatment of esophageal cancer since the esophagus is positioned close to vital structures, such as the heart and lung. The volume and relative doses to these normal tissues affect acute and late term complications. Advances in radiation delivery from 2D to 3D conformal radiation therapy, to Intensity Modulated Radiation Therapy (IMRT) or charged particle therapy (carbon ion or proton beam therapy (PBT)), allow incremental improvements in the therapeutic ratio. This could have implications in non-cancer related morbidity for long term survivors. This article reviews the evolution in radiation technologies and the use of PBT with chemotherapy in the management of esophageal cancer.

## 1. Trimodality Therapy is a Standard of Care for the Treatment of Esophageal Cancers

Esophageal cancer is the seventh leading cause of cancer deaths worldwide [1]. There is great heterogeneity in the incidence of esophageal cancer around the World, with incidence rates ranging from 30 to as high as 800 cases per 100,000 persons. Worldwide, squamous cell cancers constitute 95% of the pathologies seen, while adenocarcinomas are a more common entity in the Western world [2]. This largely reflects the differences in the epidemiology and the etiology of esophageal cancers in the different regions of the World.

Except for the earliest stage cancer, a multidisciplinary approach incorporating surgery, radiation, chemotherapy (“trimodality therapy”) should be employed. Worldwide, surgery is the mainstay of treatment for all stages of esophageal cancers. For all comers, the 5-year overall survival is around 20–25% [3,4]. Esophagectomy with either a tranthoracic or transhiatal approach is utilized. Transhiatal esophagectomy is less morbid, but because of the poorer exposure and limited dissection it is most appropriate for distal tumors. Transthoracic approaches, either a right thoracotomy (Ivor-Lewis) or left thoracotomy, allow a better dissection of the lymph nodes and better exposure of all levels of the esophagus, is most often employed for mid to distal esophageal regions. A major disadvantage for the transthoracic approach is the greater postoperative morbidity, and anastomatic leakage rate compared to the transhiatal approach. However, there are no differences in outcomes with these two approaches based on a number of randomized trials [5-7] and a meta-analysis [4] comparing these two surgical approaches. Unfortunately, despite the importance of surgery in the management of esophageal cancer, surgical resection alone provides poor local control of the disease, with locoregional failure rates at around 19–57% depends on the stage of disease and resection margin status.

An important component of the management of esophageal cancers is radiation therapy. For non-metastatic disease, radiation should be administered with chemotherapy, either preoperatively or definitively. While radiation alone is effective for palliative treatment to relieve bleeding and obstruction for patients with poor performance status or advanced disease, radiation as a single modality therapy is ineffective to cure esophageal cancer. Intergroup 0122 was a randomized trial comparing radiation alone to chemotherapy with radiation. This study demonstrated that at long term followup, up to 20% of patients are cured with definitive chemoradiation, compared to 0% in the radiation alone group [8,9]. Accumulating evidence has demonstrated that neoadjuvant chemoradiation added to surgery has survival benefit compared to surgical resection alone in a few randomized trials [10,11] and at least two meta-analysis [12,13], although this strategy remains controversial as many of the positive trials are limited in terms of patient accrual, questionable methodologies in the meta-analysis [14], as well as conflicting results from some trials demonstrating no benefits to preoperative chemoradiation [15,16]. The role for surgery after chemoradiation is also controversial, as the two European randomized trials failed to demonstrate an improvement in overall survival with the addition of surgery after chemoradiation [17,18]. While many retrospective reviews demonstrated improvements in survival outcomes with the addition of surgery, these non-prospective studies cannot exclude the selection bias inherent in patients selected for surgery *versus* those that undergo definitive chemoradiation. In the postoperative setting, radiation with chemotherapy should be considered in the setting of distal esophageal/GE junction tumors as supported by the Intergroup adjuvant gastric cancer trial 0116 that included a subset of these tumors (∼25%) [19].

As mentioned above, chemotherapy is a critical component in the management of esophageal cancers, particularly for curative intent when administered concurrently with radiotherapy. There are numerous combinations of chemotherapy that can be employed, but the doublet chemotherapies typically employed are a combination of a platinum drug (cisplatin, carboplatin, oxaliplatin) with fluoropyrimidine (5FU or capecitabine) or a taxane (paclitaxel or docetaxel). Irinotecan/cisplatin demonstrated no improvement in outcomes compared to the standard 5FU/cisplatin in a randomized phase II ECOG study [20]. Currently the most standard regimen is cisplatin/5FU, since it is the regimen most commonly employed in the randomized trials. Cisplatin is given every 3 weeks, with 5FU typically administered continuously or as a bolus every 3 weeks. Orally active capecitabine is often substituted for intravenous 5FU. Weekly carboplatin (AUC = 2) and paclitaxel (50 mg/m^2^), a regimen often employed for lung cancer, was used successfully in the recently reported phase III CROSS trial, demonstrating improvement in survival for patients treated with preoperative chemoradiotherapy compared to surgery alone [21]. When chemotherapy is administered alone, either preoperatively (as done in the UK/MRC trials) or in the metastatic setting, the same two drug regimen as aforementioned given at larger systemic doses can be used or a three drug regimen is typically employed. The most common ones are cisplatin/5FU based, with either docetaxel or epirubicin as the third drug [22].

## 2. Advances in Radiation Delivery: 3D *versus* IMRT

Since radiation is an important component in the management of stage II-III esophageal cancer in the Western world, the delivery of radiation therapy is an important consideration. Before the advent of computed tomography scanning (CT) for treatment planning, 2D approaches utilizing portal imaging and fluorometric techniques to crudely visualize tumor bearing areas were used to design simple treatment fields encompassing the tumor areas without the possibility of dose conformality away from normal tissues. In the current era of CT-based planning, better visualization of the normal tissue anatomy is possible during the treatment planning process. Using this anatomical information, beams could be arranged so that the radiation dose could better conform around structures while encompassing the treatment target volume. The current worldwide standard for beam arrangement is 3D conformal radiation therapy (3DCRT), where three or four beams are arranged around the target volume and weighted more heavily in the AP/PA direction in order to spare more normal lung tissue but consequently increases the dose to the heart and spinal cord. Intensity Modulated Radiation Therapy (IMRT) has been increasingly adopted by some centers as an alternative to 3DCRT in the effort to better spare the surrounding normal structures.

Several planning studies have shown the improve dose conformality and normal tissue sparing using IMRT [23-25]. Most of these studies demonstrate the advantage of IMRT over 3DCRT in improving mean lung dose (MLD) while no additional advantage was seen for the heart and liver. Nutting *et al.* [23] performed a treatment planning study in five patients with distal esophageal tumors using a 4 field-3DCRT plan and compared this to several IMRT field arrangements, from a 4- to a 9-field equispaced IMRT plans. They discovered that strategic placement of individual beams was critical, in that the 4-field IMRT plan was able to deliver comparable PTV coverage while reducing mean lung dose as compared to either the 3DCRT or the 9-field IMRT plans. Similarly, Chandra *et al.* [25] evaluated 3DCRT plans compared to IMRT for 10 patients with distal esophageal tumors. IMRT improved the V10 by 10%, V20 by 5%, and the mean lung dose by 2.5 Gy compared to 3DCRT, while there was no improvement observed for dose to the heart, liver, spinal cord, or total body integral doses.

The advantage of IMRT can also be seen for cervical esophageal sites. In another planning study by Fenkell *et al.* [24], five patients with tumors in the cervical esophageal region were chosen for planning studies comparing 3DCRT with IMRT, with escalating doses at 56, 63, and 70 Gy. The authors found IMRT improved target volume coverage with better conformality as well as decreased dose to adjacent normal structures, such as the brainstem, spinal cord, and the parotids.

## 3. Further Improving IMRT Delivery to Improve Cardiac Dosimetry

In the aforementioned planning studies comparing 3DCRT and IMRT, the key advantages of IMRT is the sparing of adjacent normal structures. In the setting of distal esophageal tumors, the greatest advantage was seen in the sparing of the lung. At MDACC, there has been a gradual evolution of IMRT technique compared to when it was first utilized in 2004 in order to optimize dosing to both lung and cardiac structures. This experience was recently published by Grosshans *et al.* [26]. To demonstrate this, treatment plans were generated for 12 patients using four different beam arrangements. These beam arrangements either utilized five beams arranged in two traditional configurations, either as a “hemispheric” arrangement entering from the left thorax or a “butterfly” formation with two beams entering anteriorly and three beams posteriorly, were compared to two plans that were designed with posterior and lateral beam entry points in order to avoid entering directly through the heart. Doses to the heart are delivered only by the lower exit doses. These new configurations, either as the “dragonfly” or the “firefly” techniques, significantly reduced mean cardiac doses by nearly 10 Gy, with the V20 and V30 reduced by almost half compared to the traditional beam arrangements. The compromise was in the lung dosing, although the increase was very slight and considered clinically acceptable, with the mean lung dose increased by 1.0–1.5 Gy compared to the butterfly technique. Because of the significantly improved cardiac dosing with only a slight increase in the lung dose, these new techniques have become the standard of care for IMRT treatment planning for esophageal cancer at MD Anderson.

## 4. The Dosimetric Advantage of Charged Particle Therapy Compared to IMRT for Esophageal Cancer

The dosimetric advantage of charged particles is due to the differing physical properties compared to photons (or X-rays). Charged particle therapy utilizes ionizing radiation, most commonly protons or carbon, to treat cancer. In contrast to photons, which enters the body and deposits the greatest amount of energy at a shallow depth below the skin surface (called Dmax) and leaving a trail of attenuated energy as the rays exits the body, charged particles gives relatively low dose at the surface but the cross-sectional probability of its interaction with the electrons in the body increases as the particle slows down in tissue, with the energy deposited at a depth that is a function of the energy and nature of the charged particle. This spike in energy deposition is known as the Bragg peak, named after William Henry Bragg who first described it in 1903. The dose immediately beyond the Bragg peak is practically zero, with very little neutrons contributing to the dose beyond the peak. This property can be exploited for therapy since the radiation dose can be concentrated within the target volume while minimizing dose to surrounding healthy tissue. Since the Bragg peak is too narrow to be clinically useful, several Bragg peaks of decreasing energies are superpositioned to create the so-called spread-out Bragg peak (SOBP) that provides a uniform dose that covers the target. While the entrance dose of the SOBP is greater than that of a single Bragg peak, it is still lower than the entrance dose from photon radiation.

Dosimetric analysis of protons has been compared to photon in several planning studies. In a study comparing protons with photons using 3D planning in five patients [27], proton plans spared better all structures (spinal cord, lung, heart and kidneys) and enhanced tumor control probability value by an average of 20%-units (range 2–23% units) using 5% NTCP in any risk organ. The dosimetric superiority of protons was not only confined to the comparison with 3DCRT radiation, but also extends to IMRT treatment planning. In a recent study, IMRT plans were compared to 2F (AP/PA) or 3F (AP/two posterior obliques) proton beam plans for 15 patients [28]. Both proton plans were able to improve lung sparing by reducing the V5, 10, and 20 Gy(RBE) and the MLD and cord sparing, but not for the heart using the beam arrangements as prescribed. A recent planning study comparing Intensity Modulated Proton Therapy (IMPT) to IMRT demonstrated further reduction in the dose to the lung, heart and liver with the proton plan [29]. These studies demonstrate the promise of protons to improve the therapeutic ratio in the treatment of esophageal cancer.

## 5. Challenges in the Use of PBT for the Treatment of Esophageal Cancer

In theory, proton therapy may be superior to photon radiation in terms of improving the therapeutic ratio. However, the property of the finite range in tissue that is the hallmark of proton therapy is also potentially problematic, especially in distal esophageal tumors where tumor motion due to respiration is a key concern. Extreme care must be taken into consideration for the need to compensate for tumor motion and changes in lung density due to respiration. While there are no reports that have directly studied this question in esophageal cancers, this has been done for lung tumors and reveals the complexities of proton planning for moving targets. Lessons learned from these studies could be applied for esophageal cancer treatment planning as well. A planning study in which three sets of plans were generated for a single patient with lung tumor were used to determine which plan provided the best target coverage; with each plan utilizing different apertures and different definitions of distal margins [10]. Because of the uncertainties in proton range due to respiration induced tumor motion, these authors proposed that the dosimetric uncertainties for each beam direction should be assessed separately so that some amount of dosimetric uncertainty is built in into the planning of each beam. In another planning study, Engelsman *et al.* studied the effect of setup uncertainties and respiratory motion on cumulative doses to the lung tumor [11]. By adding various amount of “smearing distances” by modifying the range compensator to ensure target coverage in the presence of volume uncertainties, the investigators applied combinations of potential errors due to breathing motion and setup inconsistencies in order to allow better coverage of the clinical target volume (CTV). As a consequence, this maneuver simultaneously increased the dose to normal tissue distal to the CTV. In addition to the importance of accounting for respiratory motion as a key step in the planning process, knowing which image datasets from simulation to use for treatment planning is also equally critical. Kang *et al.* used four different 4D computed tomography (CT) imaging datasets from 10 patients for proton treatment planning: (1) the average CT; (2) the free-breathing CT; (3) the maximum intensity projection CT; and (4) the average CT in which the voxels within the internal gross tumor volume GTV (iGTV; designed to account for motion) were replaced with a constant density (AVE_RIGTV) [12]. Using a 1-cm smearing parameter, the authors found that the AVE_RIGTV plans achieved the best overall target coverage and critical structure sparing, while the maximum intensity projection plan resulted in unnecessarily large treatment volumes and normal tissue dosing, and both the average and the free-breathing plans had inadequate 4D target coverage.

While motion and tissue density uncertainties can be accounted for by adding smearing margins, uncertainties related to reproducibility in patient setup and changes in tumor volume resulting from the treatment must also be considered carefully in proton treatment planning. Hui *et al.* [30] conducted a study in which weekly 4D CT scans were acquired for eight patients during 7 weeks of IMRT for lung cancer. For each patient, passive-scattering proton therapy plans were designed, and the dose distributions were recalculated at end-inspiratory and end-expiratory phases of each weekly 4D-CT scans. They found that the target CTV coverage had little change over the course of the treatment if tumor motion was accounted for during the original proton therapy planning. The take home message is that imaging during the original 4D-CT simulation may help predict the pattern of tumor motion over the course of therapy. However, the authors also found that if skin markers were used only without daily orthogonal X-ray imaging, up to 25% of the CTV could be missed over the course of therapy, but it was 9% error if daily bone registration was used. These results indicate that daily X-ray images and bony alignment should be used at a minimum for aligning the patient for daily proton treatment.

The issue of inter- and intra-fraction motion is an especially relevant issue for a mobile organ such as the distal esophagus, and this should be considered in proton treatment planning. Many studies have published the extent of intrafractional motion of GEJ tumors based on diaphragmatic motion. To account for respiratory related motion of the esophagus, 4D CT simulation should be obtained for all patients to account for intrafractional motion. To determine reproducibility for interfractional variability, daily orthogonal KV imaging to assess not only setup, but also to assess the overall depth of free breathing for the patient throughout the course of the 5 week treatment, as the depth of breathing can change for some patients and can alter the GEJ location, as we have recently reported after analyzing interfraction tumor motion using weekly 4DCT imaging for a group of esophageal cancer patients [31].

## 6. The Clinical Experience of PBT for the Treatment of Esophageal Cancer

The reported clinical experience using proton beam in the treatment of esophageal cancers is limited. All of the reports come from the University of Tsukuba, with a first reported published in 1989 on one patient treated preoperatively with proton beam without chemotherapy [32]. The same group has since published several updates [33-35], with the most recent reported for 51 patients treated between 1985 to 2005 [35]. All thirty-three patients were treated either using a combination of X-rays (median dose 46 Gy) and protons [median 36 Gy(RBE)], for a combined total dose of 80 Gy(RBE) [range 70–90 Gy(RBE)], or 18 were treated with proton beam alone [median dose 79 Gy(RBE), range 62–98 Gy(RBE)]. There were no treatment interruptions due to radiation esophagitis or hematologic toxicities for any patients. The 5-year actuarial survival for all 51 patients was 21.1% and a median survival of 20.5 months. While 22 patients (43%) remained disease free, locoregional relapse was the most common first site of failure for the 29 patients, occurring in the primary site for 17 patients, in field nodal disease in six patients, and one out-of-field nodal failure. This updated experience demonstrates the promise of proton beam dose escalation for the treatment of esophageal cancers. However, the tolerability of combining protons with chemotherapy and the efficacy of extending the treatment to adenocarcinomas, which are far more common in the United States and Western Europe, are currently unknown.

**Figure 1.**
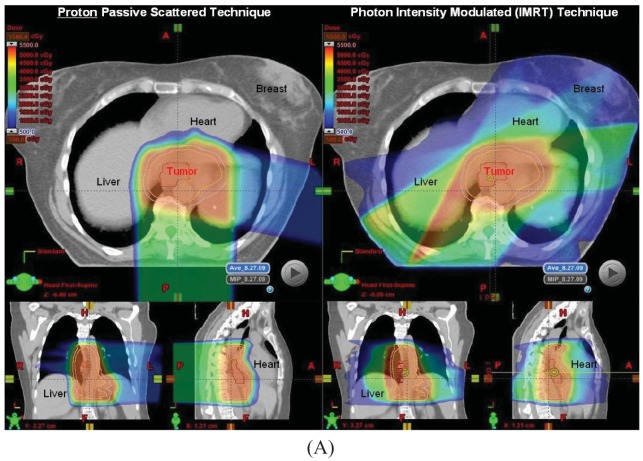

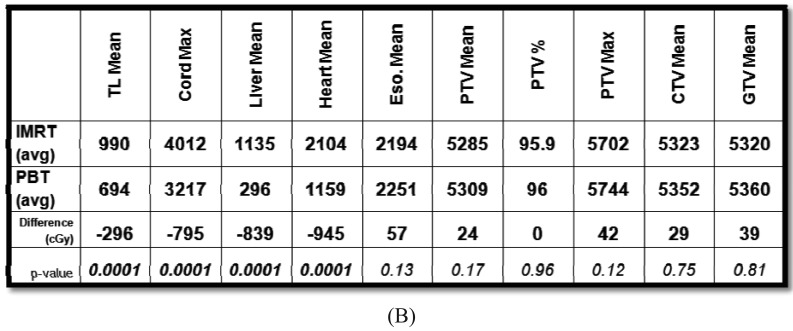
Comparison plans of IMRT and Passive Scattering Proton Beam Therapy for the treatment of distal esophageal cancer. (**A**). Dosimetric comparison of a five field modified firefly IMRT technique (right panel) with a two field passive-scattering proton plan (left panel) is made. Note the relative amount of normal tissue spared of scattered doses of radiation in the proton plan compared to the optimized IMRT plan; (**B**). Quantitative differences in the dose to normal structures and target volumes between PBT and IMRT plans in 20 matched esophageal cancer patients.

Proton beam with concurrent chemotherapy has been utilized at MD Anderson since 2006 as part of a prospective clinical trial assessing normal tissue toxicities in patients treated with protons. Our initial experience in the first 62 patients has been reported in abstract form and presented at the American Radium Society in 2011 [36]. Initially patients with larger tumors for which IMRT plans created high integral doses to the heart and lungs were selected to be treated using protons, but as our experience increased, many more patients were being treated with protons because of the improved dosimetric parameters compared to IMRT plans for the same patient in terms of heart and lung sparing, particularly for tumors at the mid to distal esophageal locations (Figure 1). All patients to date were treated with passive scattering PBT with a 1 cm smear margin for setup uncertainties. The median radiation dose was 50.4 Gy(RBE) (range 36–57.6). Most common grade 2–3 acute toxicities were dysphagia, fatigue, anorexia and nausea, and there was 1 case of grade 3 radiation pneumonitis without any grade 4–5 toxicities. Twenty-eight patients from this initial 62 patient cohort were treated preoperatively. There were a few non-fatal postoperative complications such as atrial fibrillation (N = 4), wound infection (N = 3), anastomatic leak (N = 3), and pneumonia (N = 2).

The pathologic complete response (0% viable cells) was 28%, and the near complete response (0–1% viable cells) was 50%. We have found from this initial experience on the use of PBT and chemotherapy that the treatment was well tolerated, with few severe toxicities and the pathologic response promising. A prospective randomized trial comparing PBT and IMRT is in the planning stages.

## 7. Proton Beam Treatment Planning for Esophageal Cancers at MD Anderson

We have required four-dimensional CT simulation for all patients. Patients are instructed to not eat for at least 3 hours prior to simulation, and this is to be repeated each day during treatment. Patients are immobilized with a vaccuum-lock cradle with both arms up stabilized with a T-bar. Gross tumor volume (GTV) and Clinical Tumor Volume (CTV) are delineated by the physicians, with the GTV defined by PET/CT information and endoscopy findings. Because of potential submucosal spread of disease, normal esophageal and adventitial tissue 4 cm superiorly and 1 cm radially are included in the CTV, as well as potential regional spread into the cardia of the stomach, left gastric nodal space, and celiac axis.

The treatment plan is designed based on the average CT image data set. A diaphragmatic pseudostructure is defined as the difference in lung volumes between inhaled (T0) and exhaled (T50) breathing phases. The CT number of the diaphragm is overridden with the average values from the same region on the maximum intensity projection (MIP) CT image data set. Therefore, the determined proton beam range and compensator that is designed will ensure the dose coverage at the distal end of the target volume at any phase of motion. The designed treatment plan is then applied to the T0 and T50 phase image data set without override to evaluate the dose coverage for these two extreme ends of breathing phase. We have often noticed that the dose coverage in the proximal end of the spread out Bragg peak (SOBP) on the T0 image data set is insufficient, especially for the left lateral field. The reason is that the proton beam range determined on the average CT is too deep (that is, there is an overshoot) for the target volume on the T0 phase (inhaled) where diaphragm is moved downward. This results in an under coverage of the dose distribution proximal to the SOBP covering the upper part of the target volume. To ensure dose coverage in all breathing phases, the proximal margin is then increased until adequate coverage on the T0 phase is achieved. The modified planning parameters are then used for the plan based on the average CT data set to generate the final plan. The distal and proximal margins, and smearing parameters are based on Moyers *et al.* [37].

The beam arrangements that are used largely depend on the location and extent of the target volume. We have found for most cases, a two beam plan is adequate, but an occasional three-beam approach may be optimal. For most GEJ tumors, a left lateral or a slight left posterior oblique beam entry and a posterior-anterior (PA) beam entry are adequate to cover the target volume adequately and optimally spare the lung, liver, and heart (Figure 1A). For larger volumes that extend from upper thoracic region to the GEJ, we found an anterior-posterior (AP) and PA beam arrangement was the best compromise for normal tissue sparing, particularly for the lungs, but with compromised increased dose to the heart. We have analyzed the dosimetry of the first 20 patients treated with PBT and performed optimal IMRT plans for these patients. On average and across all parameters, PBT was able to reduce dose to the liver, lung, heart and spinal cord without compromising coverage to the target volumes (Figure 1B).

Common dose constraints for photon-based radiotherapy are exercised for proton beam planning as well. For the lung, we keep the MLD < 20 Gy(RBE) and the V20 < 35%, but we make the best effort to reduce this further to under 20%, especially in preoperative cases. For the heart, we keep the V40 < 50%. For the liver, a V30 < 40% and a mean dose of <30 Gy are acceptable. For the left kidney, no greater than 1/3 of the organ should receive a dose greater than 20 Gy. If the dose exceeds this, a quantitative renal perfusion scan should be performed to assess bilateral renal functionality.

## 8. Conclusions

The incorporation of radiotherapy has remained an important part in the multi-modality management of esophageal cancer treatment in the western world. Advances in radiation delivery are capable of improving the dose conformality in order to minimize radiation to surround normal organs. Photon-based radiation is limited by the fact that photons must exit the patient, and therefore despite the use of technologies such as IMRT, dose spread is inevitable to the heart, lungs, liver, kidneys, and spinal cord. Proton beam radiation has the distinct advantage of the Bragg peak that reduces the integral dose to these normal structures, and therefore has the potential to spare patients possible postoperative and late term complications. Early reports from single institutional experiences using Protons in esophageal cancer have been encouraging. To assess the benefits of protons, future prospective studies will need to be designed to rigorously compare protons to photon-based treatments.

## References

[1] Enzinger P.C., Mayer R.J. (2003). Esophageal cancer. N. Engl. J. Med..

[2] Devesa S.S., Blot W.J., Fraumeni J.F. (1998). Changing patterns in the incidence of esophageal and gastric carcinoma in the United States. Cancer.

[3] Muller J.M., Erasmi H., Stelzner M., Zieren U., Pichlmaier H. (1990). Surgical therapy of oesophageal carcinoma. Br. J. Surg..

[4] Hulscher J.B.F., Tijssen J.G.P., Obertop H., van Lanschot J.J.B. (2001). Transthoracic *versus* transhiatal resection for carcinoma of the esophagus: A meta-analysis. Ann. Thorac. Surg..

[5] Hulscher J.B.F., van Sandick J.W., de Boer A.G.E.M., Wijnhoven B.P.L., Tijssen J.G.P., Fockens P., Stalmeier P.F.M., ten Kate F.J.W., van Dekken H., Obertop H. (2002). Extended transthoracic resection compared with limited transhiatal resection for adenocarcinoma of the esophagus. N. Engl. J. Med..

[6] Chu K.M., Law S.Y.K., Fok M., Wong J. (1997). A prospective randomized comparison of transhiatal and transthoracic resection for lower-third esophageal carcinoma. Am. J. Surg..

[7] Rentz J., Bull D., Harpole D., Bailey S., Neumayer L., Pappas T., Krasnicka B., Henderson W., Daley J., Khuri S. (2003). Transthoracic *versus* transhiatal esophagectomy: A prospective study of 945 patients. J. Thorac. Cardiovasc. Surg..

[8] Herskovic A., Martz K., Al-Sarraf M., Leichman L., Brindle J., Vaitkevicius V., Cooper J., Byhardt R., Davis L., Emami B. (1992). Combined chemotherapy and radiotherapy compared with radiotherapy alone in patients with cancer of the esophagus. N. Engl. J. Med..

[9] Cooper J.S., Guo M.D., Herskovic A., Macdonald J.S., Martenson J.A., Al-Sarraf M., Byhardt R., Russell A.H., Beitler J.J., Spencer S. (1999). Chemoradiotherapy of locally advanced esophageal cancer: Long-term follow-up of a prospective randomized trial (RTOG 85-01). J. Am. Med. Assoc..

[10] Walsh T.N., Noonan N., Hollywood D., Kelly A., Keeling N., Hennessy T.P.J. (1996). A comparison of multimodal therapy and surgery for esophageal adenocarcinoma. N. Engl. J. Med..

[11] Tepper J., Krasna M.J., Niedzwiecki D., Hollis D., Reed C.E., Goldberg R., Kiel K., Willett C., Sugarbaker D., Mayer R. (2008). Phase III trial of trimodality therapy with cisplatin, fluorouracil, radiotherapy, and surgery compared with surgery alone for esophageal cancer: CALGB 9781. J. Clin. Oncol..

[12] Gebski V., Burmeister B., Smithers B.M., Foo K., Zalcberg J., Simes J. (2007). Survival benefits from neoadjuvant chemoradiotherapy or chemotherapy in oesophageal carcinoma: A meta-analysis. Lancet Oncol..

[13] Urschel J.D., Vasan H. (2003). A meta-analysis of randomized controlled trials that compared neoadjuvant chemoradiation and surgery to surgery alone for resectable esophageal cancer. Am. J. Surg..

[14] Crehange G., Bonnetain F., Peignaux K., Truc G., Blanchard N., Rat P., Chauffert B., Ghiringhelli F., Maingon P. (2010). Preoperative radiochemotherapy for resectable localised oesophageal cancer: A controversial strategy. Crit. Rev. Oncol. Hematol..

[15] Burmeister B.H., Smithers B.M., Gebski V., Fitzgerald L., Simes R.J., Devitt P., Ackland S., Gotley D.C., Joseph D., Millar J. (2005). Surgery alone *versus* chemoradiotherapy followed by surgery for resectable cancer of the oesophagus: A randomised controlled phase III trial. Lancet Oncol..

[16] Bosset J.F., Gignoux M., Triboulet J.P., Tiret E., Mantion G., Elias D., Lozach P., Ollier J.C., Pavy J.J., Mercier M. (1997). Chemoradiotherapy followed by surgery compared with surgery alone in squamous-cell cancer of the esophagus. N. Engl. J. Med..

[17] Stahl M., Walz M.K., Stuschke M., Lehmann N., Meyer H.J., Riera-Knorrenschild J., Langer P., Engenhart-Cabillic R., Bitzer M., Königsrainer A. (2009). Phase III comparison of preoperative chemotherapy compared with chemoradiotherapy in patients with locally advanced adenocarcinoma of the esophagogastric junction. J. Clin. Oncol..

[18] Bedenne L., Michel P., Bouché O., Milan C., Mariette C., Conroy T., Pezet D., Roullet B., Seitz J.F., Herr J.P. (2007). Chemoradiation followed by surgery compared with chemoradiation alone in squamous cancer of the esophagus: FFCD 9102. J. Clin. Oncol..

[19] Macdonald J.S., Smalley S.R., Benedetti J., Hundahl S.A., Estes N.C., Stemmermann G.N., Haller D.G., Ajani J.A., Gunderson L.L., Milburn Jessup J. (2001). Chemoradiotherapy after surgery compared with surgery alone for adenocarcinoma of the stomach or gastroesophageal junction. N. Engl. J. Med..

[20] Kleinberg L., Powell M.E., Forastiere A., Keller S., Anne P., Benson A.B. (2008). Survival outcome of E1201: An Eastern Cooperative Oncology Group (ECOG) randomized phase II trial of neoadjuvant preoperative paclitaxel/cisplatin/radiotherapy (RT) or irinotecan/cisplatin/RT in endoscopy with ultrasound (EUS) staged esophageal adenocarcinoma. J. Clin. Oncol..

[21] Gaast A.V., van Hagen P., Hulshof M., Richel D., van Berge Henegouwen M.I., Nieuwenhuijzen G.A., Plukker J.T., Bonenkamp J.J., Steyerberg E.W., Tilanus H.W. (2010). Effect of preoperative concurrent chemoradiotherapy on survival of patients with resectable esophageal or esophagogastric junction cancer: Results from a multicenter randomized phase III study. J. Clin. Oncol..

[22] Ajani J.A., Barthel J.S., Bentrem D.J., D'Amico T.A., Das P., Denlinger C.S., Fuchs C.S., Gerdes H., Glasgow R.E., Hayman J.A. (2010). Esophageal and esophagogastric junction cancers. J. Natl. Compr. Cancer Netw..

[23] Nutting C.M., Bedford J.L., Cosgrove V.P., Tait D.M., Dearnaley D.P., Webb S. (2001). A comparison of conformal and intensity-modulated techniques for oesophageal radiotherapy. Radiother. Oncol..

[24] Fenkell L., Kaminsky I., Breen S., Huang S., van Prooijen M., Ringash J. (2008). Dosimetric comparison of IMRT *versus* 3D conformal radiotherapy in the treatment of cancer of the cervical esophagus. Radiother. Oncol..

[25] Chandra A., Guerrero T.M., Liu H.H., Tucker S.L., Liao Z., Wang X., Murshed H., Bonnen M.D., Garg A.K., Stevens C.W. (2005). Feasibility of using intensity-modulated radiotherapy to improve lung sparing in treatment planning for distal esophageal cancer. Radiother. Oncol..

[26] Grosshans D., Boehling N.S., Palmer M., Spicer C., Rolly E., Cox J.D., Komaki R., Chang J.Y. (2011). Improving cardiac dosimetry: Alternative beam arrangements for intensity modulated radiation therapy planning in patients with carcinoma of the distal esophagus. Pract. Radiat. Oncol..

[27] Isacsson U., Lennernäs B., Grusell E., Jung B., Montelius A., Glimelius B. (1998). Comparative treatment planning between proton and x-ray therapy in esophageal cancer. Int. J. Radiat. Oncol. Biol. Phys..

[28] Zhang X., Zhao K.L., Guerrero T.M., McGuire S.E., Yaremko B., Komaki R., Cox J.D., Hui Z., Li Y., Newhauser W.D. (2008). Four-dimensional computed tomography-based treatment planning for intensity-modulated radiation therapy and proton therapy for distal esophageal cancer. Int. J. Radiat. Oncol. Biol. Phys..

[29] Welsh J., Gomez D., Palmer M.B., Riley B.A., Mayankkumar A.V., Komaki R., Dong L., Zhu X.R., Likhacheva A., Liao Z. (2010). Intensity-modulated proton therapy further reduces normal tissue exposure during definitive therapy for locally advanced distal esophageal tumors: A dosimetric study. Int. J. Radiat. Oncol. Biol. Phys..

[30] Hui Z., Zhang X., Starkschall G., Li Y., Mohan R., Komaki R., Cox J.D., Chang J.Y. (2008). Effects of interfractional motion and anatomic changes on proton therapy dose distribution in lung cancer. Int. J. Radiat. Oncol. Biol. Phys..

[31] Wang J., Lin S.H., Dong L., Balter P., Mohan R., Komaki R., Cox J.D., Starkschall G. Quantifying the Interfractional Displacement of the Gastroesophageal Junction during Radiation Therapy for Esophageal Cancer.

[32] Shibuya S., Takase Y., Watanabe M., Orii K., Iwasaki Y., Kitagawa T. (1989). Usefulness of proton irradiation therapy as preoperative measure for esophageal cancer. Dis. Esophagus.

[33] Koyama S., Tsujii H. (2003). Proton beam therapy with high-dose irradiation for superficial and advanced esophageal carcinomas. Clin. Cancer Res..

[34] Sugahara S., Tokuuye K., Okumura T., Nakahara A., Saida Y., Kagei K., Ohara K., Hata M., Igaki H., Akine Y. (2005). Clinical results of proton beam therapy for cancer of the esophagus. Int. J. Radiat. Oncol. Biol. Phys..

[35] Mizumoto M., Sugahara S., Nakayama H., Hashii H., Nakahara A., Terashima H., Okumura T., Tsuboi K., Tokuuye K., Sakurai H. (2010). Clinical results of proton-beam therapy for locoregionally advanced esophageal cancer. Strahlenther. Onkol..

[36] Lin S.H., Komaki R., Liao Z., Wei C., Myles B., Guo X., Palmer M., Mohan R., Swisher S.G., Hofstetter W.L. Proton Beam Therapy and Concurrent Chemotherapy for Esophageal Cancer.

[37] Moyers M.F., Miller D.W., Bush D.A., Slater J.D. (2001). Methodologies and tools for proton beam design for lung tumors. Int. J. Radiat. Oncol. Biol. Phys..

